# Health-related quality of life in cancer survivors aged 65 and older: impact of diagnosis and treatment and perceived decline associated with aging

**DOI:** 10.1007/s00520-025-10139-y

**Published:** 2025-11-17

**Authors:** Yolanda Andreu, Carmen Picazo, Ana Soto-Rubio, Beatriz Gil-Julia

**Affiliations:** 1 Personality, Assessment and Psychological Treatments Department, Faculty of Psychology and Speech Therapy, University of Valencia, Valencia, Spain; 2 Psychology and Sociology Department, Faculty of Social and Human Sciences, University of Zaragoza, Teruel, Spain

**Keywords:** Cancer survivors, Older adult, Quality of life, Pain, Fatigue, Cognitive problems

## Abstract

**Purpose:**

The health-related quality of life (HRQOL) among older adult cancer survivors can be influenced by physiological and psychosocial challenges stemming from treatment and disease experience. Despite assumptions that aging might exacerbate these difficulties, research indicates higher HRQOL in this subgroup compared to younger adult cancer survivors. Objectives: (i) to analyze HRQOL in this subgroup, contrasting with younger survivors and (ii) to explore potential moderating factors related to sociodemographic and disease variables.

**Method:**

A cross-sectional study was conducted involving 655 Spanish cancer survivors aged 65 or older from several medical centers and cancer patients’ associations. HRQOL was assessed using the Quality of Life in Adult Cancer Survivors (QLACS) scale. Participants’ data was compared with data from younger survivors (*N* = 772) [8] . Two Multivariate Variance Analyses (MANOVA) were performed to address the study objectives.

**Results:**

Older adult survivors exhibited superior HRQOL across all explored domains (except family distress) compared to younger survivors. Predictors of HRQOL included age, primary cancer treatment, and time post-primary treatment. A significant interaction was noted between age and marital status.

**Conclusions:**

The observed higher HRQOL among older adult cancer survivors, particularly in domains seemingly unaffected by cancer, warrants cautious interpretation compared to their younger counterparts. In certain domains, the decline associated with aging might obscure or impede the perception of the true impact of cancer diagnosis and treatment.

## Introduction

Health programs for prevention and early cancer detection and constant medical and pharmacological advances have allowed people diagnosed and treated for cancer to live longer [1, 2]. However, their quality of life (QOL) may be affected by different late and long-term physical effects of treatment, such as pain, fatigue, and cognitive impairment [3, 4], as well as psychosocial problems related to fear of recurrence [5, 6] and difficulties in resuming social, family, and work roles [5, 7, 8]. Thus, the risk of distress and other psychological disorders is higher in cancer survivors [3].

Knowing the consequences of cancer is of great relevance to favour comprehensive care and improve the health-related quality of life (HRQOL) of cancer survivors [5, 6]. According to the European Organization for Research and Treatment of Cancer [9], the cancer survivor is a person who has already completed the primary treatment for the disease with curative intent and is free of cancer. Along the same lines, exploring possible modulators of cancer survivors’ HRQOL allows prioritizing attention on those at higher risk. Although still inconclusive, existing results so far show the existence of HRQOL moderating variables [10, 11] such as: type and number of strategies included in the primary treatment [5], age, sex, marital status [11, 12], and income level and employment status [8].

Among the variables mentioned, the results regarding age as a moderating factor mostly point to better QOL in older cancer survivors [1, 13]. Perhaps this finding is responsible to some extent for the underrepresentation of older adult cancer survivors in studies, which has been highlighted by numerous authors [2, 5, 14, 15]. However, cancer is primarily a disease of ageing. Overall cancer incidence rates rise steadily with increasing age, from less than 25 cases per 100,000 people in age groups under 20 years to about 350 per 100,000 people between the ages of 45 and 49 years, to more than 1000 per 100,000 people in age groups 60 years and older [16]. About 60% of cancers are diagnosed in older adults, and up to 60% of people living with cancer are 65 years of age or older [17, 18].

The health challenges faced by the cancer survivor may be exacerbated in the case of the older adult, where other age-related health problems such as declines in sensory function, restrictions in physical mobility, and increases in chronic disease development converge [19, 20]. The lack of information on older adult cancer survivors’ HRQOL becomes even more relevant considering that, in developed areas, there will be an increasingly ageing population with a high prevalence of cancer [2, 5].

In addition to the better HRQOL in older adult survivors when compared to younger survivors, already discussed, the limited existing studies highlight two sociodemographic variables as risk factors for greater cancer impact: being female and not having a partner [1, 21]. However, these studies have significant limitations that make it difficult to draw conclusions. For example, they jointly analyze participants who have not completed primary treatment with others who have [1]; they use small sample sizes limited to a particular hospital [21–23]; they focus primarily on breast or prostate cancer survivors [24] or specific sociocultural groups (ethnicity, socioeconomic status) [21, 25, 26]; or they explore modulatory factors in isolation, making it difficult to determine their independent association with HRQOL [6, 7, 27].

In light of the above, the present work, which follows a multicenter design, aims to increase our understanding of the impact of cancer on older adult cancer survivors’ post-treatment in two ways. First, HRQOL will be analyzed in a large and heterogeneous sample of older adult cancer survivors (65 years and older) and compared with younger survivors [8]. Second, the independent association of different sociodemographic and disease-related variables with HRQOL will be explored. In this case, the role of intragroup age is contemplated by differentiating between survivors aged 65–70 years and older than 70 years, following an oncogeriatric criterion [28].

## Method

### Participants and procedure

This cross-sectional study is part of a research project on HRQOL in adult oncology survivors in Spain. The project follows the ethical principles of the Declaration of Helsinki and was approved by the Research Ethics Committee of the Valencian Institute of Oncology Foundation (FIVO). Inclusion criteria for the general sample of participants were (i) to have been diagnosed with adult onset cancer; (ii) to present no clinically detectable evidence of cancer; and (iii) to have completed primary treatment with curative intent (surgery, radiotherapy and chemotherapy) at least one month before the time of the study (time frame of reference explored by QLACS). Exclusion criteria were (i) to present severe cognitive impairment and (ii) to have no knowledge of the Spanish language. The cancer survivors who met the inclusion criteria were invited to participate during one of their visits to a medical institution or cancer patient association. After providing written consent, the participants were interviewed by a psychologist during one of the survivors’ visits to the health centres. All of them agreed to participate and provided written consent. The current study focused on participants aged 65 years or older (*N* = 655) compared with younger cancer survivors from a previous study [8] (*N* = 772).

### Instruments

Health-related quality of life (HRQOL). The Quality of Life in Adult Cancer Survivors (QLACS) scale [29], Spanish version by [30] was used. It has 47 items and twelve domains: negative feelings, positive feelings, cognitive problems, physical pain, problems with sexual functioning, fatigue, social avoidance, financial problems, family-related distress, appearance concerns, distress over recurrence, and cancer benefits. Each domain consists of 4 items (except for family-related distress, with only 3 items; thus, the resulting score is multiplied by 1.33 to be compared with the other domains). The QLACS assesses HRQOL in the past month on a seven-point Likert scale (1 = never through 7 = always), with higher scores indicating lower HRQOL (except for positive feelings and benefits). Previous results with the Spanish version of QLACS support the excellent psychometric properties of the instrument (Cronbach’s alpha values higher than 0.70 in all domains, summary scales, and total scores) [31] as well as giving a total score on the scale, with the exclusion of the benefits-of-cancer domain [31] which, for this reason, was not included in the present study. Reliability indices obtained in this study were satisfactory (range Cronbach’s α subscales = 0.64–0.85). It should be noted that *α* = 0.64 for the problems with the sexual functioning variable is an acceptable value of internal consistency since this scale has less than 10 items [32].

### Statistical analysis

A variety of descriptive statistics were used to furnish a sociodemographic profile of the sample. A Multivariate Variance Analysis (MANOVA) was used to compare HRQOL between the subgroup under study and another subgroup of survivors younger than 65 years from the same multicenter study, whose HRQOL had been analyzed in a previous work [8]. Another MANOVA was used to explore the sociodemographic (age, gender, and marital status) and disease-related (cancer type, primary treatment, and survival phase) predictors of HRQOL in older adult cancer survivors. Due to the numerous independent variables and the likely small size of the resulting subgroups, analyses were limited to the main effect and second-order interactions. Because of the different groups’ sizes, Pillai’s trace (V) was used to evaluate the multivariate significant overall differences. Follow-up univariate F tests were conducted, and significant results on the univariate tests were followed by Bonferroni’s comparisons between all possible pairs of means. The statistical significance level for analyses was *p* < 0.05. Statistical analysis was performed using IBM SPSS Statistics, version 26.0.

## Results

Descriptive data on sociodemographic and disease-related variables and HRQOL domains are shown in Tables 1 and 2. The mean age of the participants was 71.7 (*SD* = 5.35), with 51.8% older than 70 years. Most of them were male (63.4%), married or living with a partner (75.9%), and did not have a university education (80.9%). The distribution of cancer type was prostate (32.3%), colorectal (22.7%), breast (18.4%), melanoma (4.7%), head and neck (4.5%), multiple (6.9%), and others (10.5%).

**Table 1:** Characteristics of the participants (*N* = 655)

**Variable**		***n* (%)**
Age (years)	65–70	316 (48.2)
x̅ = 71.7, *SD* = 5.4 (65–92)	> 70	339 (51.8)
Sex	Female	239 (36.6)
	Male	414 (63.4)
Marital status	With Partner	490 (75.9)
	Without Partner	156 (24.1)
Level of studies	Without studies	102 (16.6)
	Primary education	297 (48.4)
	Secondary education	98 (16.0)
	University studies	117 (19.1)
Cancer type	Prostate	207 (32.3)
	Colorectal	145 (22.7)
	Breast	118 (18.4)
	Head & neck	29 (4.5)
	Melanoma	30 (4.7)
	Multiple	44 (6.9)
	Others (hematological, lung, gastric, etc.)	67 (10.5)
Primary treatment	S, RT, or S + RT	412 (64.3)
	CT with or without S	93 (14.5)
	CT + RT with or without S	136 (21.2)
Survival phase (months)	RES	123 (19.3)
x̅ = 79.2; *SD* = 64.5 (1—360)	ES	254 (39.9)
	LTS	259 (40.7)

Note:* S*, surgery; *RT*, radiotherapy; *CT*, chemotherapy; *RES*, re-entry survivorship (≤ 12); *ES*, early survivorship (13–59); *LTS*, long-term survivorship (≥ 60)

**Table 2:** Internal consistency, means, and standard deviations (in brackets) for domains HRQOL and distress for the total sample of cancer survivors (*N* = 1.437) and the two sub-groups by age: < 65 (*N* = 772) and ≥ 65 (*N* = 655) and MANOVA results

Variable	Mean (*SD*) < 65 years	Mean (*SD*) ≥ 65 years	*F*
Domains HRQOL (α) *V* =.121; *F* = 17.550; df_between_ = 11; df_error_ = 1398; *p* ≤.001			
Negative feelings (*α* =.72)	12.75 (5.06)	10.61 (4.78)	53.238***
Positive feelings (*α* =.80)	20.29 (5.60)	21.67 (5.56)	14.378***
Cognitive problems (*α* =.71)	11.41 (6.22)	9.51 (4.79)	45.230***
Sexual problems (*α* =.64)	12.50 (6.62)	10.91 (5.87)	11.702***
Pain (*α* =.83)	11.34 (6.22)	8.95 (5.43)	41.011***
Fatigue (*α* =.81)	12.88 (5.84)	10.86 (5.45)	34.975***
Socialavoidance (*α* =.85)	8.60 (5.18)	8.23 (4.48)	40.789***
Appearance concerns (*α* =.82)	10.52 (6.50)	6.68 (4.39)	148.316***
Financial problems (*α* =.77)	7.47 (5.30)	4.95 (2.80)	83.262***
Distress-recurrence (*α* =.78)	14.42 (6.60)	11.79 (6.49)	53.250***
Distress-family (*α* =.83)	13.06 (5.87)	12.37 (6.49)	2.731

Note: ***p ≤.001; α: Cronbach’s α; *V*, Pillai’s Trace value; *F*, Fisher´s *F* value

Regarding primary treatment with curative intent, more than half of the participants (64.3%) had received local treatment; 14.5% had received systemic treatment (with or without surgery), and 21.2% had received combined treatment with radiotherapy and chemotherapy (with or without surgery). Lastly, the average length of time elapsed after the completion of primary treatment was 79.16 months (*SD* = 64.50) (range: 1 month–30 years). Concerning time of survival, the study follows the proposal of Stanton et al. [33] that distinguishes three survival phases. Specifically, 19.3% of participants had ended primary treatment in the previous 12 months (re-entry survivorship phase, RES); 40.7% had completed it at least five years before the interview (long-term survivorship phase, LTS), and 39.9% had exceeded 12 months after primary treatment but had not yet reached five years (early survivorship phase, ES) (Table 1).

The MANOVA results for the comparison of HRQOL between the subgroup under study and the subgroup of cancer survivors younger than 65 years were significant (*p* ≤ 0.001), indicating better HRQOL in the older adult subgroup in all domains (*p* ≤ 0.001 in all cases), except for family distress (see Table 2).

Results regarding the prediction of HRQOL are shown in Tables 3 and 4. Predictors of HRQOL were primary treatments (*p* ≤ 0.05), survival phase (*p* ≤ 0.001), and age (*p* ≤ 0.05). A single interaction effect between age and marital status was found to be significant (*p* ≤ 0.05) (see Table 3).

**Table 3:** MANOVA factorial (7^a^ × 3^b^ × 3^c^ × 2^d^ × 2^e^ × 2^f^) domains of HRQOL

Source of variation	*V*	*F*	df_between_	df_error_
(A) Cancer type	.207	1.260	66	2328
**(B) Primary treatments**	**.089**	1.630*	**22**	**768**
**(C) Survival phase**	**.111**	2.044***	**22**	**768**
**(D) Age**	**.057**	2.124*	**11**	**383**
(E) Gender	.040	1.457	11	383
(F) Marital status	.038	1389	11	383
(A X B) Cancer type* Primary treatments	.179	.720	99	3519
(A X C) Cancer type* Survival phase	.302	.926	132	433
(A X D) Cancer type*Age	.163	.985	66	2328
(A X E) Cancer type*Gender	.143	1.039	55	1935
(A X F) Cancer type*Marital status	.201	1.225	66	2328
(B XC) Primary treatments*Survival phase	.092	.825	44	1544
(B X D) Primary treatments*Age	.071	.983	22	768
(B XE) Primary treatments*Gender	.042	.745	22	768
(B X F) Primary treatments*Marital status	.062	1.110	22	766
(C X D) Survival phase*Age	.079	1.429	22	768
(C X E) Survival phase*Gender	.067	1.213	22	768
(C X F) Survival phase*Marital status	.057	1.024	22	768
(D X E) Age*Gender	.030	1.065	11	383
(D X F) Age*Marital status	**.058**	2.136*	**11**	**383**
(E X F) Gender*Marital status	.021	.744	11	383

Note: *p ≤.05, **p ≤.01, ***p ≤.001; *V*, Pillai’s Trace value; *F,* Fisher´s *F* value; *df*, degrees of freedom

**Table 4:** Means, standard deviations (in brackets), *F* values, and post hoc Bonferroni procedure for Domains HRQOL by Primary treatments, Survival Phase, Age, Age*Marital Status

**Primary treatment**	**Type 1**	**Type 2**	**Type 3**		***F***
Cognitive problems	9.10 (4.58)	10.06 (4.66)	10.36 (5.36)		5.135**
**Survival phase**	**RES**	**ES**	**LTS**		***F***
Positive feelings	20.73 (5.97)^c^	21.58 (5.57)	22.40 (5.14)^a^		5.669**
Pain	9,64 (5.70)	8.64 (5.31)	8.97 (5.38)		7.106***
Fatigue	11.63 (5.83)	11.06 (5.51)	10.44 (5.26)		8.285***
Social Avoid	8.69 (4.89)	8.33 (4.74)	7.98 (4.05)		3.863*
**Age**	** ≤ 70 years**	** > 70 years**			***F***
Pain	9.19 (5.75)	8.72 (5.11)			4.084*
Fatigue	10.79 (5.42)	10.93 (5.49)			3.939*
Age*Marital Status	** ≤ 70 years**		** > 70 years**		***F***
	Without partner	With partner	Without partner	With partner	
Positive feelings	20.36 (5.88)^g^	21.96 (5.51)^f,g^	19.89 (5.84)^e^	22.41 (5.26)^d,e^	7.455**

Note: Only significant results are shown in the table

^*^p ≤.05, **p ≤.01, ***p ≤.001

Type 1 = S, RT, or S + RT, Type 2 = CT with or without S, and Type 3 = CT + RT with or without S

The superscripts indicate significant differences with respect to: ^a^RES; ^b^ES; ^c^LTS; ^d^without a partner up to 70; ^e^without a partner over 70; ^f^with a partner up to 70; ^g^with a partner over 70

Survival phase (months): *RES*, re-entry survivorship (≤ 12), *ES*, early survivorship (13–59); *LTS*, long-term survivorship (≥ 60)

Participant age was associated with the pain and fatigue domains (*p* ≤ 0.05 in both cases), indicating that participants older than 70 years had less pain and more fatigue (see Table 4).

The interaction effect between age and marital status was statistically significant in the positive feelings domain (*p* ≤ 0.01). Those over 70 years of age with a partner showed greater positive feelings than younger both married and single people. Those older than 70 years without a partner showed the lowest score in the domain, although the difference was significant only with the younger married subgroup (see Table 4).

Differences by type of primary treatment were concentrated in the domain of cognitive problems (*p* ≤ 0.01). The means corresponding to the different subgroups pointed to more significant problems when the treatment was not exclusively local (see Table 4). However, post hoc contrasts showed no significant differences between subgroups.

The survival phase was associated with the domains of positive feelings (*p* ≤ 0.001), pain (*p* ≤ 0.001), fatigue (*p* ≤ 0.001), and social avoidance (*p* ≤ 0.05). In all cases, the data pointed to an improvement with increasing time after the end of treatment. However, only the domain of positive feelings showed significant differences between subgroups, indicating a greater presence of positive feelings in the long survival phase versus the re-entry phase (see Table 4).

## Discussion

The first objective of the present study was to analyze the HRQOL of older adult cancer survivors. As a reference criterion for its assessment, we compared the results with those obtained previously in a subgroup of survivors under 65 years of age [8]. The results indicated better HRQOL in older adult survivors than in younger survivors in all domains, except for family distress. Given the age-associated decline in physical functioning [19, 34], these results may at first be surprising. However, they are consistent with what has been reported in previous research [11, 35]. The more disruptive nature of the cancer experience in younger people, posing a greater threat and impact on their personal and professional development, along with less life experience and learning, has been the argument frequently put forward to justify this result [1, 8, 11]. Conversely, older adult cancer survivors may view cancer as just another “bump in the road” [1].

Concern about the possibility of cancer in a family member was the only outcome not associated with age. Such concern is congruent with findings from different investigations of cancer survivors [3, 5, 11]. That it is not moderated by age may derive from the first-person experience of the severe and prolonged impact of cancer diagnosis and treatment. Moreover, regardless of age, the theoretical risk of cancer becomes a reality, increasing the perception of vulnerability of oneself and also of one’s loved ones.

The second objective of the present study was to analyze the moderating role of different sociodemographic and disease-related variables in the HRQOL of older adult cancer survivors.

Among the sociodemographic variables, age played a relevant role both at the level of the main effect as well as at the level of interaction effects in the prediction of HRQOL. The greater fatigue and less pain reported by older adult cancer survivors find support in the literature addressing the effects of the ageing process. Age-related changes in muscle and cardiovascular/pulmonary functions would be a possible cause of the fatigue relatively prevalent in the general older population [36]. Similarly, age-related changes in nociceptive processing may account for the lower pain perception and reporting observed among older adults [15, 27].

The interaction of age with marital status in predicting HRQOL showed the importance of having a partner in those over 70 years of age at the level of positive affectivity. Research results have consistently supported the critical role in the cancer experience of a partner as a key source of social support [37, 38]. Our results show that, at least at the affective level, the role played by the partner is even more relevant in the case of older adult cancer survivors. Particularly susceptible to social and emotional loneliness due to various factors associated with their life stage [35, 39], the benefit of having a partner would be even greater. The companionship and social support provided by a partner would mitigate such feelings of loneliness and contribute to a greater extent to their emotional well-being [7, 39].

Among the disease-related variables, it is noteworthy that, unlike what was found in younger cancer survivors [1, 8], the type of cancer did not have a statistically significant effect on the HRQOL of older adult cancer survivors. However, to a limited extent, the primary treatment protocol did impact the HRQOL of the older adult cancer survivor. The results of different studies indicate that the greater complexity entailed by the treatment is associated with a greater deterioration of HRQOL [4, 5, 11, 40]. Specifically, the presence of chemotherapy in the treatment protocol affects cognitive ability, pain, and fatigue [40, 41]. The inclusion of radiotherapy alongside chemotherapy results in even greater side effects, particularly in terms of fatigue and cognitive problems [15, 42]. Nonetheless, the greater impact of primary treatment, as its complexity increases, was observed among older adult cancer survivors only in the cognitive domain.

The survival phase, the third of the disease-related variables, was the one that showed the most relevant role. Consistent with what was found in the literature [1], a longer time since the end of primary treatment was associated with decreased pain and fatigue. Likewise, social avoidance and positive affectivity also improved over time. The improvement described in symptomatology (fatigue and pain) may be responsible, to some extent, for the improvement found in these functional aspects. A decrease in symptoms would translate into greater well-being while facilitating contact with friends and participation in social gatherings [43, 44]. At the same time, greater well-being and social participation would also have a reciprocal effect between them, mutually increasing each other.

The results described above are striking in that older adult cancer survivors report improvement over time in aspects of HRQOL that have not been impacted by cancer diagnosis and treatment, which leads us to the following reflection. HRQOL is a subjective assessment of the impact of a disease on an individual’s quality of life [5, 8, 15]. As Weiner’s (1985) [45] attributional theory postulates, expected outcomes promote stable attributions, whereas unexpected outcomes facilitate unstable attributions. Thus, the assessment of the impact of cancer on their HRQOL may be affected in older adult cancer survivors due to perceived decline being attributed to age instead of to cancer/treatment effect. In this regard, many conditions that would not be considered normal in a younger population are routinely accepted in older people as a part of so-called “normal” ageing [15, 26]. It would be the case, for example, that many older adults feel that pain is “just a normal part of ageing” [27] and, as we have already noted, fatigue is relatively prevalent in the general older population [35]. Pain and fatigue are precisely two of the most relevant and prevalent side effects experienced by cancer survivors [1, 5, 22]. However, neither of these symptoms was reported by the older adult survivor as impacted by the type or treatment of cancer.

The presence of pain and fatigue associated with the changes experienced with age could be masking or making it difficult to perceive the impact of the cancer and consequently attribute it a role in these symptoms. Cognitive problems, another frequent side effect of cancer treatment [4, 39], were instead associated in older adult survivors with cancer treatment. There is also an ageing-related loss in cognitive reserve capacities; however, many changes in cognitive functions occur throughout life and are not necessarily linked to old age [36]. It is possible that the longer perceived cognitive decline may be facilitating the attribution of the impact of cancer in this regard.

In summary, the obtained results raise the possibility that, in certain domains, the improved HRQOL observed in older adult cancer survivors may stem from a potential bias or misjudgment in assigning the role of aging as a primary cause for the experienced impairment in their quality of life. Thus, caution seems to advise that, before assuming that middle-aged cancer survivors should be targeted (vs. older adult) in efforts to improve HRQOL [1], it would be advisable to delve deeper into this issue. Given the cross-sectional design of the present work, future longitudinal studies addressing within-subject changes in HRQOL experienced by older adult survivors over time after completion of primary treatment would be of interest. The QLACS instructions merely ask the respondent to indicate the changes experienced in HRQOL in the last four weeks. Thus, it would also be of interest for future studies to inquire in a differentiated manner about, on the one hand, the deficits experienced in HRQOL and, on the other hand, to what these deficits are attributed to: cancer or ageing.

This study’s strengths included exploring the HRQOL in a large sample of older adult survivors and comparing it with another large sample of younger survivors, both of which were integrated into the same Spanish National Public Health System. The exploration of HRQOL was performed through the QLACS, which was created specifically for the assessment of QOL in cancer survivors and has demonstrated good psychometric properties. We analyzed the role of age within the older adult survivor group by considering those older than 70 years versus those younger (65–70) [28]. Finally, we analyzed the independent association of possible sociodemographic and disease-related variables on the HRQOL of older adult survivors.

## Data Availability

No datasets were generated or analysed during the current study.
